# Stage-dependent fate of *Plasmodium falciparum*-infected red blood cells in the spleen and sickle-cell trait-related protection against malaria

**DOI:** 10.1186/s12936-016-1522-0

**Published:** 2016-09-21

**Authors:** Seidina A. S. Diakité, Papa Alioune Ndour, Valentine Brousse, Frederick Gay, Camille Roussel, Sylvestre Biligui, Michaël Dussiot, Virginie Prendki, Tatiana M. Lopera-Mesa, Karim Traoré, Drissa Konaté, Saibou Doumbia, Jérôme Cros, Safi Dokmak, Rick M. Fairhurst, Mahamadou Diakité, Pierre A. Buffet

**Affiliations:** 1 INSERM U1134, Paris 5, Paris 7, Institut National de la Transfusion Sanguine, 75015 Paris, France; 2 Malaria Research and Training Center, Faculty of Medicine, Pharmacy and Odontostomatology, University of Bamako, Bamako, BP 1805 Mali; 3 Laboratoire d’Excellence du Globule Rouge (GR-Ex), 75115 Paris, France; 4 Centre de Référence de la Drépanocytose, Hôpital Universitaire Necker Enfants Malades, 75012 Paris, France; 5 INSERM U1163/CNRS ERL 8254, Laboratory of Cellular and Molecular Mechanisms of Hematological Disorders and Therapeutic Implications, Institut Imagine, Université Paris Descartes-Sorbonne Paris Cité, Paris, France; 6 Laboratory of Malaria and Vector Research, National Institute of Allergy and Infectious Diseases, National Institutes of Health, Rockville, MD 20852 USA; 7 Department of Chirurgie Digestive et Viscérale, Hôpital Beaujon, AP-HP, 92110 Clichy, France

**Keywords:** Malaria, *Plasmodium falciparum*, Sickle-cell trait, Red blood cell, Spleen, Retention

## Abstract

**Background:**

Sickle-cell trait (HbAS) reduces falciparum malaria risk and suppresses parasitaemia. Although several candidate mechanisms have been proposed, their epidemiological, clinical and experimental correlates have not been adequately explained. To explore the basis for generally lower parasitaemias and delayed malaria episodes in children with HbAS, it is hypothesized here that their spleen-dependent removal of ring-infected red blood cells (RBCs) is more efficient than in children with normal haemoglobin A (HbAA).

**Methods:**

The mechanical splenic retention of *Plasmodium falciparum*-infected RBCs from subjects with HbAS or HbAA was investigated using two physiologically relevant methods: microsphiltration and ex vivo spleen perfusion. *P. falciparum*-infected RBCs obtained from in vitro cultures and from patients were used in either normoxic or hypoxic conditions. The effect of sickling in ring-infected HbAS RBCs was also investigated.

**Results:**

When a laboratory-adapted parasite strain was analysed, ring-infected HbAA RBCs were retained in microsphilters at similar or greater levels than ring-infected HbAS RBCs, under normoxic (retention rate 62.5 vs 43.8 %, *P* < 0.01) and hypoxic (54.0 vs 38.0 %, *P* = 0.11) conditions. When parasitized RBCs from Malian children were analysed, retention of ring-infected HbAA and HbAS RBCs was similar when tested either directly ex vivo (32.1 vs 28.7 %, *P* = 0.52) or after one re-invasion in vitro (55.9 vs 43.7 %, *P* = 0.30). In hypoxia, sickling of uninfected and ring-infected HbAS RBCs (8.6 vs 5.7 %, *P* = 0.51), and retention of ring-infected HbAA and HbAS RBCs in microsphilters (72.5 vs 68.8 %, *P* = 0.38) and spleens (41.2 vs 30.4 %, *P* = 0.11), also did not differ. Retention of HbAS and HbAA RBCs infected with mature *P. falciparum* stages was greater than 95 %.

**Conclusions:**

Sickle-cell trait is not associated with higher retention or sickling of ring-infected RBCs in experimental systems reflecting the mechanical sensing of RBCs by the human spleen. As observed with HbAA RBCs, HbAS RBCs infected with mature parasites are completely retained. Because the cytoadherence of HbAS RBCs infected with mature parasites is impaired, the very efficient splenic retention of such non-adherent infected RBCs is expected to result in a slower rise of *P. falciparum* parasitaemia in sickle-cell trait carriers.

**Electronic supplementary material:**

The online version of this article (doi:10.1186/s12936-016-1522-0) contains supplementary material, which is available to authorized users.

## Background

Despite its deleterious consequences, haemoglobin S (HbS, β_6_ Glu → Val) has reached high prevalence in malaria-endemic areas [1, 2] due to its natural selection by falciparum malaria. HbS exists as a balanced polymorphism in sub-Saharan Africa, where it protects children with sickle-cell trait (HbAS heterozygotes) from falciparum malaria morbidity and mortality but causes sickle-cell disease in HbSS homozygotes [3]. While HbAS children are protected from severe malaria, uncomplicated malaria, and high parasitaemia [4–8] compared to HbAA children, the mechanisms that mediate these protections remain under active investigation. Although several candidate mechanisms have been proposed [9–11], their epidemiological, clinical and experimental correlates have not been adequately explained. For example, Malian HbAS children experience their first malaria episode about 1 month later in the transmission season compared to HbAA children, despite these two groups of children having a comparable parasitaemia at the time of their first malaria episode [12].

To explore the basis for generally lower parasitaemias and delayed malaria episodes in HbAS children, it is hypothesized here that their spleen-dependent removal of ring-infected red blood cells (RBCs) is more efficient than in HbAA children. Such an effect would achieve a lower parasitaemia that would be tolerated without symptoms, and delay progression to a higher parasitaemia that produces symptoms [13, 14]. A slower rise in parasitaemia in HbAS children may conceivably result from an enhanced adaptive immune response to *P. falciparum* antigens or from a more effective innate clearance of circulating infected RBCs in the spleen, as suggested by enhanced phagocytosis of ring-infected HbAS RBCs in vitro [15]. While several studies have reported no differences between HbAS and HbAA children in the intensity of adaptive immune responses to parasite antigens [16–18], others have reported some differences [19].

In exploring a protective role for the spleen, one potentially relevant observation is that HbAS RBCs are less deformable than HbAA RBCs, evidenced in part by their greater retention in leukocyte-depletion filters [20]. Also, *P. falciparum* ring-infected HbAS RBCs were found to sickle faster than uninfected HbAS RBCs when treated with reducing agents or exposed to very low O_2_ concentrations [21, 22]. Given these findings, investigators have long speculated that the spleen’s preferential destruction of sickled, ring-infected HbAS RBCs may protect HbAS children from clinical malaria and high parasitaemia. Alternatively, since the cytoadherence of HbAS RBCs infected with mature parasites is significantly impaired [23, 24], the splenic retention and destruction of such non-adherent infected RBCs may result in a slower rise of *P. falciparum* parasitaemia in sickle-cell trait carriers. Retention of infected RBCs in the spleen may involve both biomechanical processes and cell–cell adhesion between infected RBCs and splenic structures. However, consistent with the absence of detectable parasite proteins on the surface of ring-infected RBCs, their retention in isolated-perfused spleens (performed in the absence of plasma or antibodies) is biomechanical in nature [25].

Taking advantage of recent progress in studying splenic retention in vitro and ex vivo [26], these possibilities were investigated using validated tools, such as ektacytometry, microsphiltration and human spleen perfusion under conditions that induce or do not induce the sickling of uninfected and infected HbAS RBCs.

## Methods

### Blood collection

HbAA and HbAS RBCs were collected in Mali and France. In Mali, blood donors were children who participated in a cohort study of genetic and acquired protection from falciparum malaria [5], and were found to be healthy and aparasitaemic at the time of blood collection. This study is registered at Clinicaltrials.gov (NCT00669084) and was approved by the Institutional Review Board (IRB) of the National Institute of Allergy and Infectious Diseases, US National Institutes of Health, and the Ethics Committee of the Faculty of Medicine, Pharmacy and Odontostomatology, University of Bamako. The parents of all children gave written informed consent.

In France, HbAS and HbSS RBCs were collected at the Necker Hospital from HbAS parents or HbSS children who were identified through routine haemoglobinopathy screening and prenatal counselling programmes; in all cases, parents provided written informed consent for the use of left-over blood samples. HbAA RBCs were collected from healthy donors in parallel. These sample collections were approved by the IRB of Paris VI University. The Ile de France VI IRB has approved this approach as a non-research process (Article L1121-1, French Code for Public Health).

All venous blood samples were collected in ACD Vacutainers® (Becton–Dickinson, Rungis, France) and washed three times with RPMI-1640 (Life Technologies, Saint-Aubin, France) to remove the buffy coat and plasma. RBCs were stored at 4 °C and used within 24 h.

### *Plasmodium falciparum* strains and culture

Trophozoite stages of the *P. falciparum* laboratory line FUP and Malian *P. falciparum* clinical isolates (prepared by collecting ring stages from children with malaria and culturing them ex vivo) were purified using MACS columns (Miltenyi Biotec, San Diego, CA, USA), inoculated into fresh HbAA, HbAS and HbSS RBCs in parallel, and then studied at the ring stage. In some experiments, parasites were allowed to mature for 36 h to the trophozoite stage before being used in filtration experiments.

In France, parasites were cultured at 2 % haematocrit in RPMI-1640 media containing 25 mM HEPES, 25 mM NaHCO_3_, 0.3 g/L glutamine, 10 mg/L gentamicin (Life Technologies) and 10 % human AB+ serum, and incubated at 37 °C in an atmosphere of 21 % O_2_, 5 % CO_2_ and 74 % N_2_. In Mali, parasites were cultured similarly, except that 0.5 % AlbuMAX® II (Life Technologies) was used to supplement the media instead of human AB+ serum.

### Microsphilter preparation

Calibrated metal microbeads (microspheres) composed of 96.5 % tin, 3.0 % silver and 0.5 % copper were used to reconstitute very narrow spaces mimicking the inter-endothelial slit of the human spleen red pulp micro vein (sinus). Microspheres were obtained from *Industrie des Poudres Sphériques* (24A, rue de la Résistance-BP 438, Annemasse 74108, France). Two different size distributions (5- to 15-μm diameter and 15- to 25-μm diameter) were used throughout. A total of 1 g of dry microspheres of each sort was mixed and then suspended in 5 mL of phosphate-buffered saline (PBS)/1 % AlbuMAX II (Invitrogen). A total of 800 μL of this microsphere suspension was poured into an inverted 1000-μL anti-aerosol pipette tip (Neptune, BarrierTips) and allowed to settle, leading to the formation of a 5-mm thick microsphere layer above the anti-aerosol filter.

### Microsphiltration

A total of 600 µL of RBC suspension (2 % haematocrit in PBS/1 % AlbuMAX® II) was instantaneously introduced upstream from the microsphere layer. The microsphere layer was then washed with 6 mL of PBS/1 % AlbuMAX II using an electric pump (Syramed μsp6000, Arcomed’Ag) at a flow rate of 60 mL/h. The downstream sample (6.6 mL) and an aliquot from the upstream sample were analysed. In some experiments, hypoxic conditions were created by gassing the RBC suspension and the PBS/1 % AlbuMAX® II solution with a gas mixture of 1 % O_2_, 5 % CO_2_ and 94 % N_2_ for 15 min and microsphiltration was performed in a hermetic plastic tent (Captair Pyramid®; Erlab, Val de Reuil, France) that was inflated, deflated and re-inflated with the same gas mixture prior to each experiment (Additional file 1). Maintenance of 1 % O_2_ and 5 % CO_2_ levels in the RBC suspension was confirmed every 15–30 min using an i-STAT® System and CG4+ cartridge (Abbott Point of Care, Princeton, NJ, USA). The mean parasitaemias (per cent of infected RBCs in the RBC suspension) in the upstream (U) and downstream (D) samples were determined for duplicate samples. The RBC retention rate (RR) for each sample was calculated using the following formula: RR = [(U−D)/U] × 100.

### Cell counting

Uninfected and ring-infected RBCs were labelled with SYTO® 61 red fluorescent nucleic acid stain (Life Technologies) to detect parasite DNA. Briefly, 100 µL of RBCs were incubated in 1 mL of a 1/1000 dilution of SYTO61 in PBS/2 % AlbuMAX solution for 15 min. The RBCs were then washed three times with PBS and PKH26 (red) or PKH67 (green) lipophilic fluorescent cell linker probes were used to label HbAS and HbAA RBC membranes, respectively, according to the manufacturer’s instructions (Sigma-Aldrich, Saint-Quentin, France). Cell counting was performed using an Accuri C6 flow cytometer (Becton–Dickinson), and 20,000 events were recorded.

### Sickling in stringent hypoxic conditions followed by microsphiltration

To investigate the influence of hypoxia-induced sickling on the retention of ring-infected RBCs, these cells were suspended at 2 % haematocrit in PBS/1 % AlbuMAX® II and exposed to a gas mixture containing a lower O_2_ concentration (0.5 % O_2_, 5 % CO_2_ and 94.5 % N_2_) supplemented with 2 L of CO_2_ within a hermetic plastic tent. The final concentration of CO_2_ in the tent was 6.5 %. The variation of dissolved CO_2_ and O_2_ partial pressures was measured using an i-STAT® System and CG4+ cartridge (Abbott Point of Care, Princeton, NJ, USA). At 0, 5, 15, 30 and 45 min after exposure to the gas mixture, 500-µL samples were fixed in 500 µL of 1 % glutaraldehyde for up to 15 min, pelleted and used to prepare a thin smear on a glass slide. The slide was then labelled with 1:1500 Hoechst 33,342 stain (H3570; Molecular Probes, Inc, Eugene, OR, USA) in PBS/1 % AlbuMAX® II for 30 min, washed four times with 1X PBS, and covered with VECTASHIELD® anti-fade mounting media for fluorescence (Vector Laboratories, Burlingame, CA, USA) and a cover slide. Using a fluorescence microscope, RBCs were observed under red fluorescence and parasite DNA under UV fluorescence. Images were acquired on a Leica DMI3000 microscope, using a Leica DFC310FX camera controlled by LAS Superposition Images software (Leica Microsystems, Nanterre, France). Uninfected RBCs and ring-infected RBCs were separately categorized into three groups: discoid RBCs, sickled RBCs, or indeterminate RBCs that were neither discoid nor sickled. To validate cell counts by microscopy, discoid RBCs, sickled RBCs, discoid ring-infected RBCs, and sickled ring-infected RBCs were also quantified using imaging flow cytometry as previously described [27]. Briefly, glutaraldehyde-fixed RBCs were passed through an Image Stream Mark II Imaging Flow Cytometer (EMD Millipore, Billerica, MA, USA) and 50,000 events acquired using Inspire V4.0 software. Post-acquisition data analysis was performed using IDEAS V6.2 software. An algorithm that correctly differentiates discoid and sickled RBCs was created. Sickling rates were calculated by dividing the number of sickled RBCs by all RBCs (i.e., discoid + sickled + indeterminate RBCs).

### Human spleen retrieval and ex vivo spleen perfusion

Human spleens were retrieved from patients undergoing splenopancreatectomy for pancreatic diseases and processed as previously described [28]. Briefly, medical and surgical care was not modified, and written informed consent was obtained from all patients. The study was approved by the Ile-de-France II IRB. The main splenic artery was cannulated, and the spleen flushed with RPMI-1640 containing 0.3 % Albumax II and 1 µg/L gentamicin (Life Technologies), transferred to the laboratory, and connected to the perfusion device. Fresh HbAA and HbAS RBCs were inoculated with mature-stage MACS-purified *P. falciparum* and incubated for 12–36 h at 37 °C in an atmosphere of 21 % O_2_ and 5 % CO_2_ until reinvasion occurred. The two RBC populations were then differentially labelled with two lipophilic fluorescent cell-linker probes: PKH26 for HbAA RBCs and PKH67 for HbAS RBCs. The two labelled RBC suspensions were then mixed with fresh RBCs (French Blood Establishment, Rungis, France) to achieve a 15–25 % haematocrit. The mixture was then perfused into the spleen ex vivo using the perfusion device. During perfusion, key physiologic markers (TO_2_, TCO_2_, glucose, lactate) were maintained at physiological levels. Spleen effluents were sampled at various time points during perfusion. Fluorescence signals of the HbAA RBC and HbAS RBC populations were detected and the parasitaemia of each RBC population determined by flow cytometry as described above. The relative retention rates were then calculated as described above. Relative retention rate is defined here as retention of ring-infected HbAA RBCs in all (infected and uninfected) HbAA RBCs from the same culture or retention of ring-infected HbAS RBCs in all (infected and uninfected) HbAS RBCs from the same culture (see Additional files 2, 3). The retention rate at the end of the perfusion (at 60 min) was considered the retention rate in the spleen.

### Statistical analysis

Statistical analysis was performed using GraphPad Prism 6 software (GraphPad, San Diego, CA, USA). The Mann–Whitney test, Kruskal–Wallis test, or t test with Welch’s correction were used, as appropriate. All *P* values are two-tailed and deemed significant when <0.05.

## Results

### Retention of *Plasmodium falciparum* ring-infected HbAA and HbAS RBCs

**Fig. 1:**
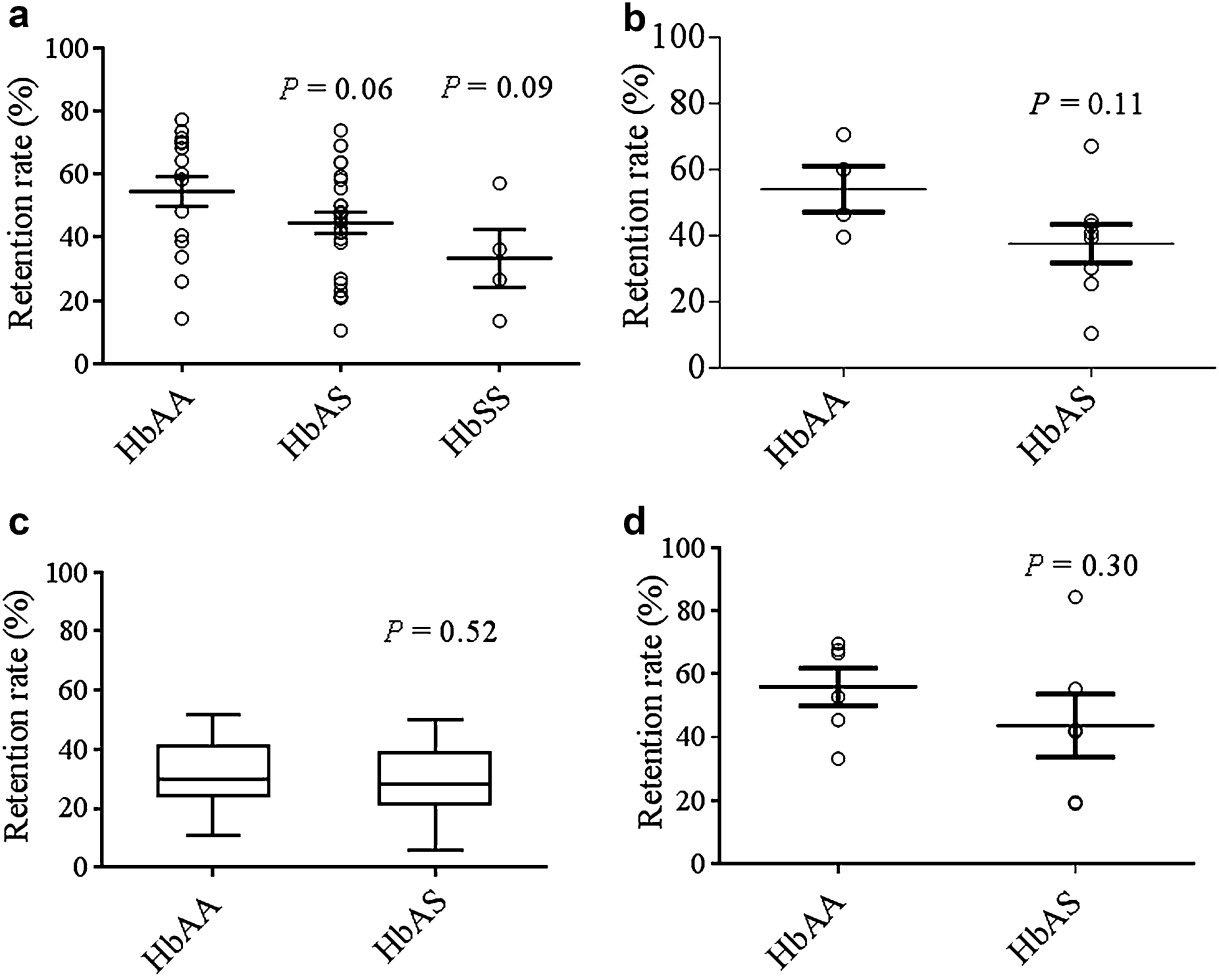
Retention of *Plasmodium falciparum* ring-infected RBCs. **a** Mean retention rate (± SEM) of *P. falciparum* (FUP line) ring-infected HbAA (n = 16), HbAS (n = 26) and HbSS (n = 4) RBCs in microsphilters under normoxic conditions. **b** Mean retention rate (± SEM) of *P. falciparum* (FUP line) ring-infected HbAA (n = 4) and HbAS (n = 8) RBCs in microsphilters exposed to ‘moderate’ hypoxia (1 % O_2_, 3 % CO_2_, 96 % N_2_ for 15 min). **c** Median retention rate (interquartile range, range) of ring-infected RBCs obtained directly from Malian HbAA (n = 29) and HbAS (n = 8) children with *P. falciparum* malaria and passed through microsphilters under normoxic conditions. **d** Mean retention rates (± SEM) of ring-infected HbAA (n = 6) and HbAS RBCs (n = 6) passed through microsphilters under normoxic conditions. These samples were prepared by obtaining *P. falciparum* isolates from Malian children with malaria, cultivating them to the schizont stage, purifying and inoculating them into fresh HbAA and HbAS RBCs simultaneously, and cultivating them to the ring stage in parallel

To mimic the situation occurring in human subjects, the relative retention rates of infected HbAA RBCs vs all (infected and uninfected) HbAA RBCs, or HbAS RBCs vs all (infected and uninfected) HbAS RBCs, were determined. In experiments using the *P. falciparum* line FUP, the mean retention rate (± SEM) of ring-infected HbAA RBCs in microsphilters was higher than that of ring-infected HbAS RBCs in both normoxic (54.5 % ± 4.7 vs 44.5 % ± 3.3, *P* = 0.06; Fig. 1a) and moderately hypoxic conditions (54.0 % ± 7.0 vs 38.0 % ± 6.0, *P* = 0.11; Fig. 1b). The mean retention rate of ring-infected HbSS RBCs in microsphilters (33.3 % ± 9.1, *P* = 0.09) was even lower in normoxic conditions (Fig. 1a). In experiments using ring-infected RBCs collected directly from Malian children with malaria, the mean retention rates of HbAA and HbAS samples were not different (32.1 % ± 2.0 vs 28.7 % ± 4.7, *P* = 0.52; Fig. 1c) (Additional file 5: Table S1). After invasion of *P. falciparum* clinical isolates into fresh HbAA and HbAS RBCs and cultivation to ring stages in vitro, the mean retention rates of HbAA and HbAS samples were also not significantly different (55.9 % ± 6.0 vs 43.7 % ± 10.0, *P* = 0.30; Fig. 1d) (Additional file 5: Table S2). When co-perfused through two isolated human spleens for 60 min ex vivo, the retention rates of ring-infected HbAA and HbAS RBCs were not significantly different (37.2 vs 45.2 % in spleen 1, and 27.7 vs 33.1 % in spleen 2) (Fig. 3a). These data indicate that ring-infected HbAS RBCs are no less filterable than ring-infected HbAA RBCs in microsphilters in vitro (even in moderately hypoxic conditions) or in human spleens ex vivo.

### Hypoxia-induced sickling of ring-infected and uninfected RBCs

**Fig. 2:**
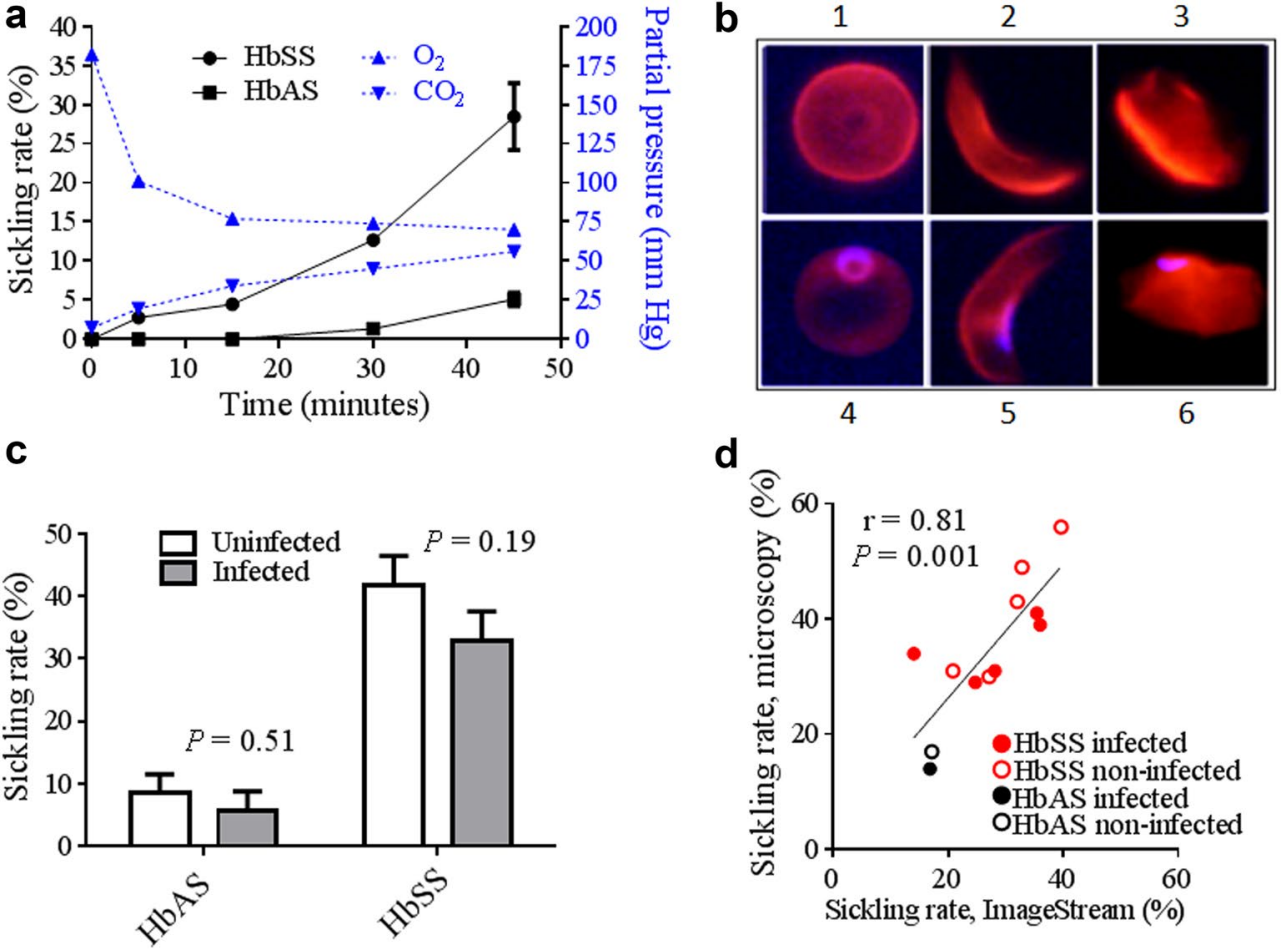
Hypoxia-induced sickling of uninfected and *Plasmodium falciparum* (FUP line) ring-infected RBCs. **a** Mean sickling rates (± SEM) in uninfected HbAS (n = 5) and HbSS RBCs (n = 3) exposed to ‘stringent’ hypoxia (0.5 % O_2_, 5 % CO_2_, 94.5 % N_2_) (*left y-axis*), and partial pressures of O_2_ and CO_2_ (*right y-axis*) measurements over 45 min. **b** Morphologies of uninfected RBCs and ring-infected RBCs (iRBCs) exposed to stringent hypoxia: discoid RBC (1), sickled RBC (2), indeterminate RBC (3), discoid iRBC (4), sickled iRBC (5) and indeterminate iRBC (6). **c** Mean sickling rates (± SEM) of uninfected and ring-infected HbAS and HbSS RBCs exposed to stringent hypoxia for 45 min. RBC morphology was determined by microscopy. **d** Correlation between mean sickling rates obtained using two RBC morphology counting methods, microscopy and imaging flow cytometry. The haemoglobin type and infection status of each sample is shown

Previous reports had observed the enhanced sickling of ring-infected HbAS RBCs compared to uninfected HbAS RBCs upon exposure to 8 % sodium metabisulfite, dithionite, or stringent hypoxic conditions (100 % N_2_) [21, 22], and speculated that this property would enhance the destruction of sickled ring-infected RBCs in the spleen. To investigate whether sickling influences the splenic retention of ring-infected RBCs, it was first established that exposure to stringent hypoxia (0.5 % O_2_, 5 % CO_2_ and 94.5 % N_2_ for 45 min) induced sickling in uninfected HbAS and HbSS RBCs (Fig. 2a). In experiments using uninfected and ring-infected RBCs (see later), sickling rates were calculated by enumerating the numbers of discoid, sickled, and indeterminate RBCs (Fig. 2b) and then calculating the proportion of all RBCs that was sickled. When exposed to stringent hypoxia for 15 min (initiated sickling) and 45 min (established sickling), no enhancement of sickling in ring-infected HbAS or HbSS RBCs was observed as compared to uninfected ring-infected HbAA RBCs. In fact, sickling rates at 45 min were slightly lower in ring-infected vs uninfected RBCs: mean ± SEM, 5.7 % ± 6.0 vs 8.6 % ± 5.7 in HbAS, and 32.7 % ± 12.0 vs 41.7 % ± 8.6 in HbSS samples (Fig. 2c). Sickling rates derived from microscopy or imaging flow cytometry were significantly correlated (Pearson r = 0.81, *P* = 0.001; Fig. 2d).

### Effect of sickling-inducing hypoxia on the retention of ring-infected HbAA and HbAS RBCs

**Fig. 3:**
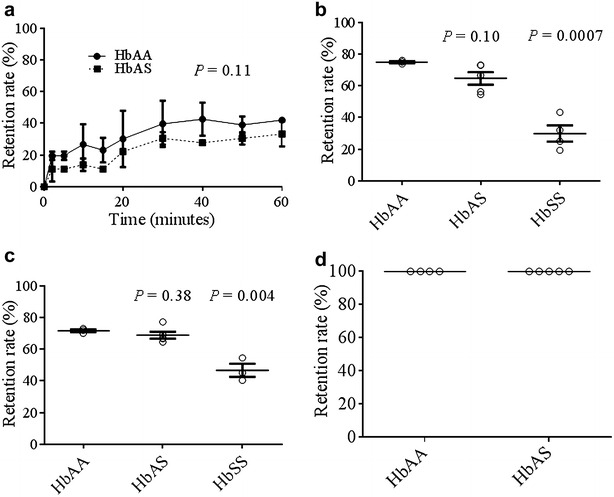
Retention of hypoxia-exposed *Plasmodium falciparum* (FUP line)-infected RBCs. **a** Mean retention rates (± SEM) of ring-infected HbAA (n = 2) and HbAS (n = 2) RBCs in two isolated human spleens over 60 min of perfusion ex vivo. **b** Mean retention rates (± SEM) of ring-infected HbAA (n = 3), HbAS (n = 5) and HbSS (n = 4) RBCs in microsphilters after exposure to ‘stringent’ hypoxia (0.5 % O_2_, 5 % CO_2_, 94.5 % N_2_) for 15 min. **c** Mean retention rates (± SEM) of ring-infected HbAA (n = 3), HbAS (n = 5) and HbSS (n = 3) RBCs in microsphilters after exposure to stringent hypoxia for 45 min. **d** Mean retention rate (± SEM) of mature *P. falciparum*-infected HbAA (n = 4) and HbAS (n = 5) RBCs in microsphilters

To investigate the effects of hypoxia-induced sickling on the splenic retention of ring-infected RBCs, *P. falciparum* (FUP line) ring-infected HbAA and HbAS RBCs were exposed to stringent hypoxia for various times and then passed through microsphilters under the same hypoxic condition. This showed that the relative retention rates of ring-infected HbAA and HbAS RBCs in microsphilters were similar, but significantly higher than those of ring-infected HbSS RBCs, after 15 min of hypoxia: mean ± SEM, 74.8 % ± 1.1 for HbAA, 64.6 % ± 8.8 for HbAS, and 29.2 % ± 10.3 for HbSS (Fig. 3b). Similar results were found after 45 min of hypoxia: 72.5 % ± 1.5 for HbAA, 68.8 % ± 4.9 for HbAS, and 46.6 % ± 7.1 for HbSS (Fig. 3c).

### Retention of mature *Plasmodium falciparum*-infected HbAA and HbAS RBCs

HbAA and HbAS RBCs were inoculated with *P. falciparum*, cultured in vitro for 36 h until parasites matured to the trophozoite stage and filtered. In four independent experiments, essentially all of the mature *P. falciparum*-infected HbAS and HbAA RBCs were retained in the microsphilters (Fig. 3d).

## Discussion

This study shows that ring-infected HbAS RBCs displayed retention rates that were similar or lower than those of ring-infected HbAA RBCs in vitro (microsphiltration) and ex vivo (perfused human spleens). To mimic the situation occurring in human subjects, experiments were performed with HbAA rings mixed in HbAA RBCs or HbAS rings mixed in HbAS RBCs. This finding was unexpected given that uninfected HbAS RBCs are less deformable than uninfected HbAA RBCs [13, 14] (Additional file 4). It was thus conceivable that, after being invaded by *P. falciparum* and subjected to the effects of initial parasite development, ring-infected HbAS RBCs would become even less deformable than uninfected HbAS RBCs and thereby more retained in microsphilters and human spleens. Despite this logical assumption, ring-infected HbAS RBCs were not more retained than ring-infected HbAA RBCs in microsphilters. This observation, which was initially made using the *P. falciparum* FUP strain in normoxic conditions, was reproduced in microsphilters using the FUP strain in moderately hypoxic conditions (1 % O_2_, 3 % CO_2_ and 96 % N_2_) and multiple parasite isolates from Malian children in normoxic conditions either before or after in vitro cultivation.

Together, these data suggest that sickle-cell trait does not suppress parasitaemia by enhancing the mechanical retention of ring-infected RBCs in the spleen. While the retention of ring-infected RBCs was partial in these experimental systems that mimic the mechanical sensing of RBCs by the spleen, mechanical retention of HbAS RBCs infected with mature parasites was very effective and complete, as previously observed with *P. falciparum*-infected HbAA RBC [25, 26]. Because the cytoadherence of HbAS RBCs infected with mature parasites is impaired [23], it is proposed here that the very efficient splenic retention of such non-adherent infected RBCs may result in a slower rise of *P. falciparum* parasitaemia in sickle-cell trait carriers.

The aforementioned experiments assumed that the microsphilter retention of ring-infected RBCs results from parasite-induced alterations in the shape or membrane rigidity of ring-infected RBCs [14, 29]. However, increased rigidity of ring-infected HbAS RBCs may also result from parasite-induced sickling, which increases their cytoplasmic viscosity. Previous studies of HbAS RBCs ex vivo [21] and in vitro [22] reported enhanced sickling of ring-infected RBCs as compared to uninfected RBCs, and thus speculated that HbAS (but not HbAA) children benefitted from enhanced destruction of ring-infected RBCs in their spleens. To test this possibility, the mechanical retention of ring-infected HbAS (and HbSS) RBCs was investigated in experimental conditions that induced sickling. When exposed to sickling-inducing, physiologically-relevant gas mixtures, both uninfected and ring-infected HbAS and HbSS RBCs showed no differences in their sickling rates. Even under conditions that induced high sickling rates, the retention rates of ring-infected HbAS and HbAA RBCs were similar. These results, which were obtained at O_2_ and CO_2_ concentrations observed in the venous effluent of human spleens perfused ex vivo [28] and without reducing agents, differ strikingly from previous observations [21, 22] and are in agreement with pioneering explorations on this topic [30]. Together, these data suggest that sickle-cell trait does suppress parasitaemia by enhancing the retention of sickled, ring-infected RBCs in the spleen.

**Fig. 4:**
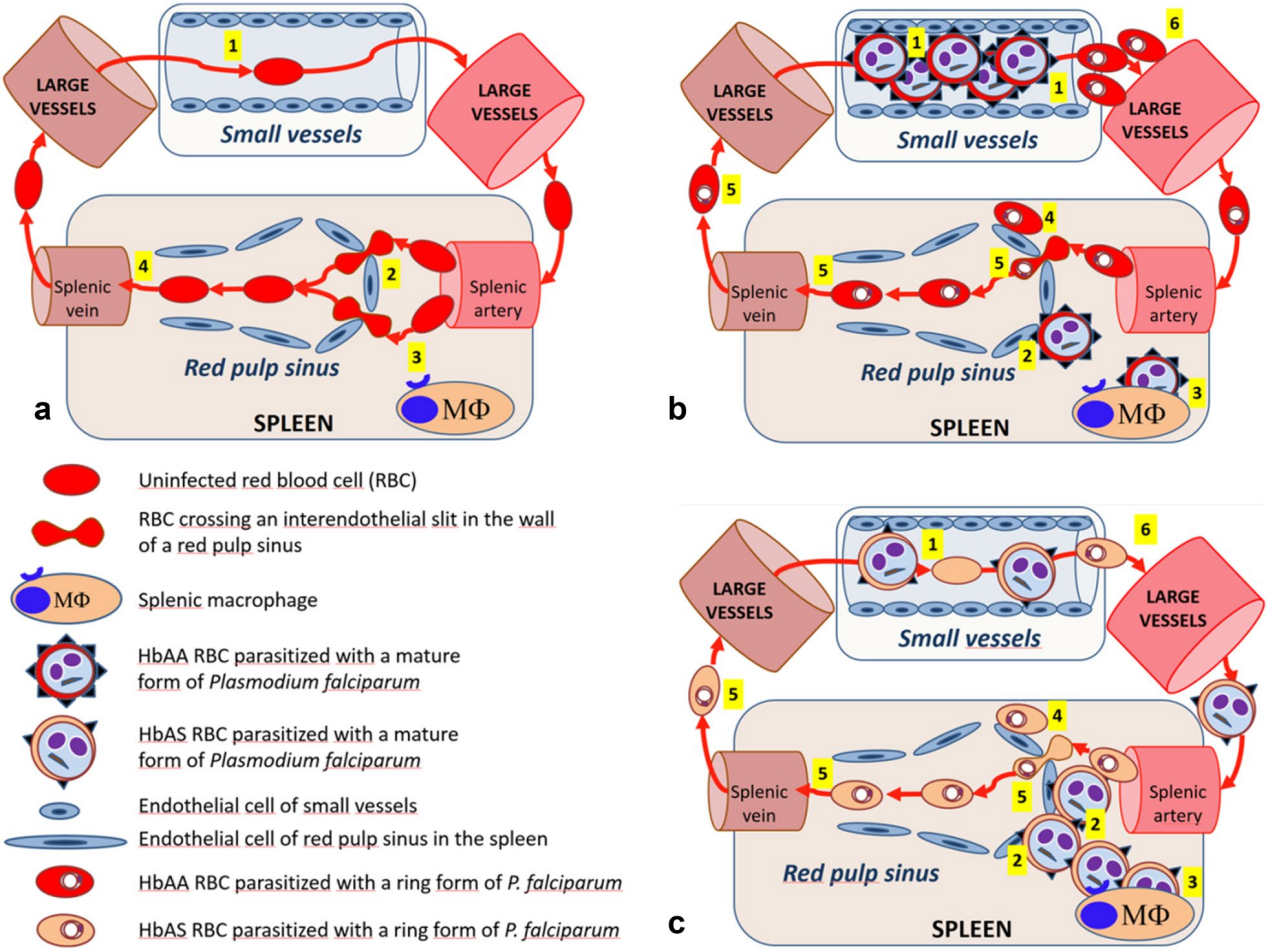
Proposed model for sickle-cell trait-related protection from high *Plasmodium falciparum* parasitaemia and severe malaria. **a** The preserved deformability and normal surface of RBCs enable their efficient navigation through small vessels *1*, crossing of narrow inter-endothelial slits (IES) in the wall of red pulp sinuses in the spleen *2*, and escape from recognition by red pulp macrophages (MΦ) *3*, resulting in rapid exiting from the spleen *4*. **b** In HbAA patients, mature *P. falciparum*-infected RBCs express numerous PfEMP1 adhesins on their surface that mediate their accumulation in small vessels through adherence to endothelial cells *1*. The few rigid mature forms that escape cytoadherence-based sequestration in small vessels cannot cross IES in the spleen *2*, and are ultimately phagocytosed by splenic macrophages *3*. While a proportion of RBCs infected with young ring forms is mechanically retained upstream from IES *4*, most persist in circulation *5*. The large population of RBCs infected with mature forms sequestered in small vessels *1* causes direct pathogenic effects leading to severe malaria and rapidly leads to high parasitaemia *6*. **c** In HbAS patients, mature *P. falciparum*-infected RBCs express few PfEMP1 adhesins on their surface, resulting in a less-intense accumulation in small vessels *1*. Those RBCs that escape cytoadherence-based sequestration in small vessels cannot cross IES in the spleen *2*. This retention is expected to result in their phagocytosis by splenic macrophages *3*. The small population of HbAS RBCs infected with mature forms sequestered in small vessels *1* give rise to only a small population of circulating ring-infected RBCs *6*. HbAS patients may be protected from severe malaria by an amelioration of pathogenic effects due to a smaller biomass of infected RBCs sequestered in small vessels resulting from mechanical retention of non-adherent RBCs infected with mature forms in the spleen. This mechanism also explains lower parasitaemia and delayed time to a malaria episode

Overall, these results suggest that the malaria-protective effects of HbAS do not involve enhanced retention of ring-infected RBCs in the spleen, and call for a re-analysis of proposed mechanisms of protection that implicate parasite-induced sickling in clearing ring-infected HbAS RBCs or impairing parasite development in HbAS RBCs [17]. This study shows that mature *P. falciparum*-infected HbAS RBCs are very efficiently mechanically retained in the spleen. These findings reinforce other candidate mechanisms of malaria protection, for example, abnormal PfEMP1 display and impaired cytoadherence of trophozoite-infected HbAS RBCs [23]. By reducing the proportion of mature trophozoite-infected RBCs that is bound to the microvessels of vital organs, HbAS may increase the proportion that is retained and destroyed in the spleen, thus suppressing parasitaemia in the next life cycle and ameliorating the symptoms of uncomplicated and severe malaria (Fig. 4). This model of malaria protection is supported by previous findings that HbAS is associated with a 50 % reduction in the binding of mature trophozoite-infected RBCs to microvascular endothelial cells [23] and that mature trophozoite-infected RBCs are completely retained in microsphilters and isolated perfused human spleens [26]. These data are supported by this study’s finding that all mature trophozoite-infected HbAS RBCs are essentially retained in microsphilters.

## Conclusions

These observations suggest a very important role of the spleen in controlling parasite load in HbAS children by the efficient retention and phagocytosis of those mature trophozoite-infected RBCs that fail to cytoadhere [15].

## Additional files

10.1186/s12936-016-1522-0 The hermetic plastic tent and gas tank connection used for hypoxia-induced sickling of uninfected and ring-infected RBCs.
10.1186/s12936-016-1522-0 Schematic representation of the microsphiltration procedure using RBCs infected in vitro with (A) a *Plasmodium falciparum* laboratory strain (FUP) or *P. falciparum* clinical isolates, or (B) naturally infected RBCs from patients.
10.1186/s12936-016-1522-0 Schematic representation of the ex vivo spleen perfusion procedure.
10.1186/s12936-016-1522-0 Elongation indices of freshly collected uninfected HbAA, HbAS and HbSS RBCs measured by ektacytometry.
10.1186/s12936-016-1522-0 Characteristics of Malian patients from whom ring-infected RBCs were collected for ex vivo microsphiltration.** Table S2.** Characteristics of Malian patients from whom *Plasmodium falciparum* clinical isolates were used in in vitro reinvasion and microsphiltration experiments.
