# Supplementary figures and images for: Stage-dependent fate of Plasmodium falciparum-infected red blood cells in the spleen and sickle-cell trait-related protection against malaria
Source: Malar J. 2016 Sep 21;15:482. doi: 10.1186/s12936-016-1522-0 (PMC5031340; doi:10.1186/s12936-016-1522-0)

**Additional file 1**


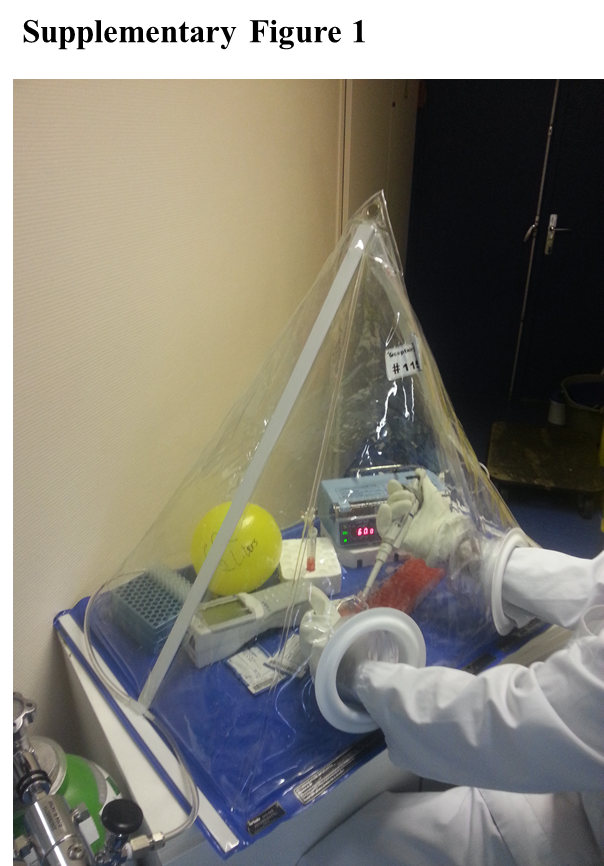

Supplement: Supplementary file 1 — 10.1186/s12936-016-1522-0 The hermetic plastic tent and gas tank connection used for hypoxia-induced sickling of uninfected and ring-infected RBCs. [file 12936_2016_1522_MOESM1_ESM.docx]

**Additional file 2**


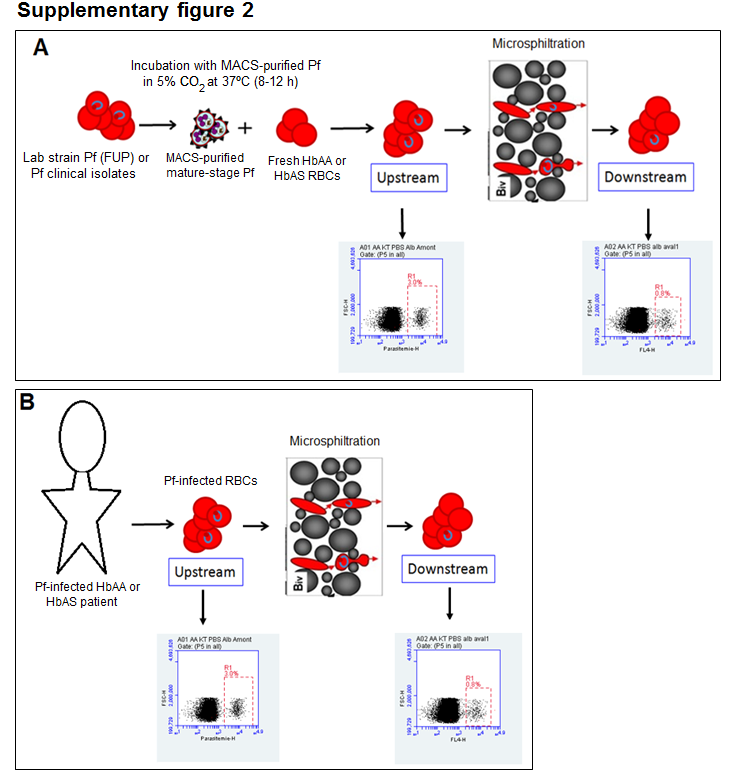

Supplement: Supplementary file 2 — 10.1186/s12936-016-1522-0 Schematic representation of the microsphiltration procedure using RBCs infected in vitro with (A) a Plasmodium falciparum laboratory strain (FUP) or P. falciparum clinical isolates, or (B) naturally infected RBCs from patients. [file 12936_2016_1522_MOESM2_ESM.docx]

**Additional file 3**


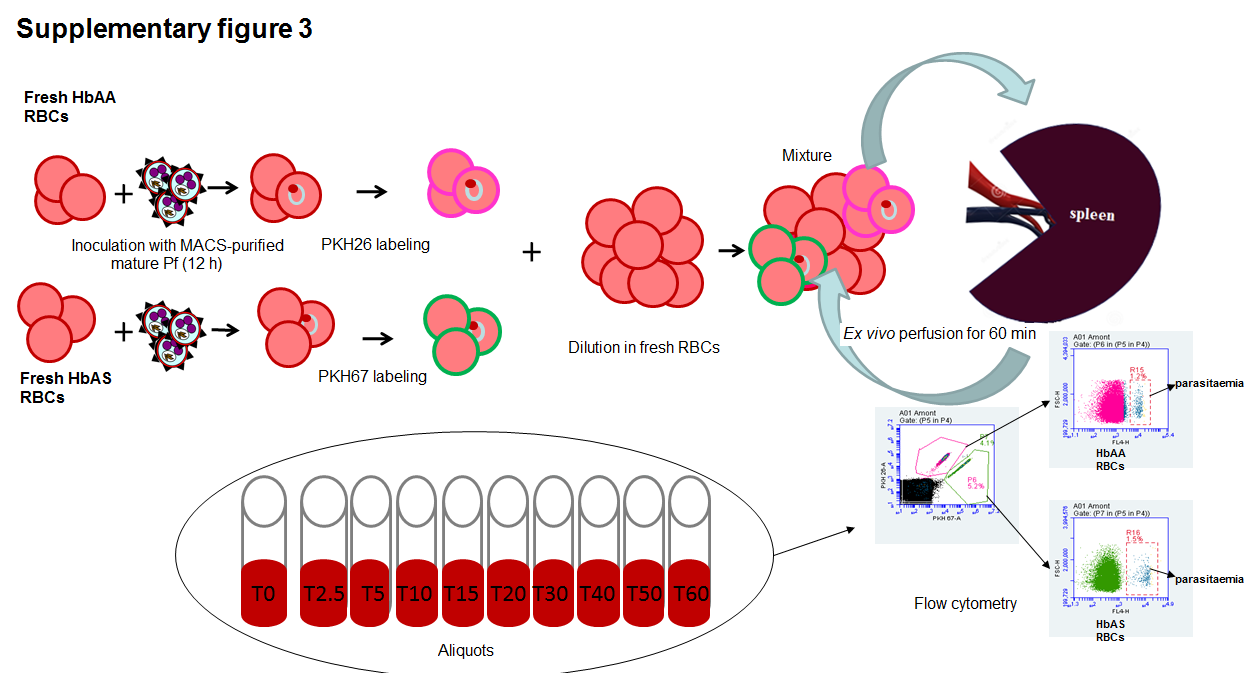

Supplement: Supplementary file 3 — 10.1186/s12936-016-1522-0 Schematic representation of the ex vivo spleen perfusion procedure. [file 12936_2016_1522_MOESM3_ESM.docx]

**Additional file 4**


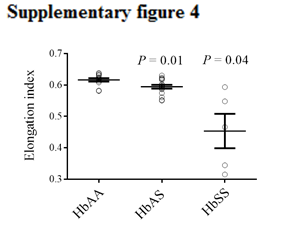

Supplement: Supplementary file 4 — 10.1186/s12936-016-1522-0 Elongation indices of freshly collected uninfected HbAA, HbAS and HbSS RBCs measured by ektacytometry. [file 12936_2016_1522_MOESM4_ESM.docx]
