# Supplementary material for: Stage-dependent fate of Plasmodium falciparum-infected red blood cells in the spleen and sickle-cell trait-related protection against malaria
Source: Malar J. 2016 Sep 21;15:482. doi: 10.1186/s12936-016-1522-0 (PMC5031340; doi:10.1186/s12936-016-1522-0)
Supplement: Supplementary file 5 — 10.1186/s12936-016-1522-0 Characteristics of Malian patients from whom ring-infected RBCs were collected for ex vivo microsphiltration. Table S2. Characteristics of Malian patients from whom Plasmodium falciparum clinical isolates were used in in vitro reinvasion and microsphiltration experiments. [file 12936_2016_1522_MOESM5_ESM.docx]

**Table S1.**

| **Sample** | **Age** | **Gender** | **Blood group** | **Haemoglobin type** | **α-thalassaemia status (3.7-kb deletion)** |
| --- | --- | --- | --- | --- | --- |
| 1 | 5 | F | A | AA | Wild type |
| 2 | 3 | F | O | AA | Wild type |
| 3 | 6 | M | B | AA | Heterozygous |
| 4 | 8 | F | O | AA | Wild type |
| 5 | 5 | M | A | AS | Wild type |
| 6 | 4 | F | A | AA | Wild type |
| 7 | 3 | M | O | AA | Heterozygous |
| 8 | 6 | M | O | AA | Wild type |
| 9 | 3 | M | B | AS | Wild type |
| 10 | 2 | F | B | AA | Wild type |
| 11 | 4 | M | O | AA | Wild type |
| 12 | 5 | M | A | AS | Heterozygous |
| 13 | 4 | M | O | AA | Wild type |
| 14 | 6 | F | B | AS | Wild type |
| 15 | 9 | M | O | AA | Wild type |
| 16 | 15 | M | A | AA | Wild type |
| 17 | 3 | M | A | AS | Wild type |
| 18 | 5 | F | O | AA | Heterozygous |
| 19 | 9 | M | O | AA | Wild type |
| 20 | 4 | M | O | AA | Wild type |
| 21 | 12 | M | B | AA | Wild type |
| 22 | 5 | M | A | AA | Wild type |
| 23 | 4 | F | A | AS | Wild type |
| 24 | 6 | F | O | AA | Heterozygous |
| 25 | 4 | M | B | AA | Wild type |
| 26 | 7 | F | O | AA | Wild type |
| 27 | 5 | M | O | AA | Wild type |
| 28 | 6 | M | A | AA | Wild type |
| 29 | 4 | M | B | AA | Wild type |
| 30 | 3 | F | O | AA | Wild type |
| 31 | 5 | F | A | AA | Wild type |
| 32 | 10 | M | O | AA | Wild type |
| 33 | 4 | F | O | AA | Wild type |
| 34 | 8 | M | B | AS | Wild type |
| 35 | 3 | F | O | AA | Wild type |
| 36 | 5 | M | AB | AA | Wild type |
| 37 | 3 | M | O | AS | Wild type |

**Table S2:**

| **Sample** | **Age** | **Gender** | **Blood group** | **Haemoglobin type** | **α-thalassaemia status (3.7-kb deletion)** |
| --- | --- | --- | --- | --- | --- |
| 3 | 6 | M | B | AA | Heterozygous |
| 6 | 4 | F | A | AA | Wild type |
| 14 | 6 | F | B | AS | Wild type |
| 16 | 15 | M | A | AA | Wild type |
| 19 | 9 | M | O | AA | Wild type |
| 32 | 10 | M | O | AA | Wild type |
